# Transcriptomic analysis delineates potential signature genes and miRNAs associated with the pathogenesis of asthma

**DOI:** 10.1038/s41598-020-70368-5

**Published:** 2020-08-07

**Authors:** Prithvi Singh, Archana Sharma, Rishabh Jha, Shweta Arora, Rafiq Ahmad, Arshad Husain Rahmani, Saleh A. Almatroodi, Ravins Dohare, Mansoor Ali Syed

**Affiliations:** 1 Centre for Interdisciplinary Research in Basic Sciences, Jamia Millia Islamia, New Delhi, 110025 India; 2 Translational Research Lab, Department of Biotechnology, Faculty of Natural Sciences, Jamia Millia Islamia, New Delhi, 110025 India; 3 Centre for Nanoscience and Nanotechnology, Jamia Millia Islamia, New Delhi, 110025 India; 4 Department of Medical Laboratories, College of Applied Medical Sciences, Qassim University, Buraydah, 51452 Saudi Arabia

**Keywords:** Regulatory networks, Statistics, Data mining, Gene ontology, Gene regulatory networks, Microarrays, Network topology, Proteome informatics, Software, Statistical methods, Asthma, Biotechnology, Developmental biology

## Abstract

Asthma is a multifarious disease affecting several million people around the world. It has a heterogeneous risk architecture inclusive of both genetic and environmental factors. This heterogeneity can be utilised to identify differentially expressed biomarkers of the disease, which may ultimately aid in the development of more localized and molecularly targeted therapies. In this respect, our study complies with meta-analysis of microarray datasets containing mRNA expression profiles of both asthmatic and control patients, to identify the critical Differentially Expressed Genes (DEGs) involved in the pathogenesis of asthma. We found a total of 30 DEGs out of which 13 were involved in the pathway and functional enrichment analysis. Moreover, 5 DEGs were identified as the hub genes by network centrality-based analysis. Most hub genes were involved in protease/antiprotease pathways. Also, 26 miRNAs and 20 TFs having an association with these hub genes were found to be intricated in a 3-node miRNA Feed-Forward Loop. Out of these, miR-34b and miR-449c were identified as the key miRNAs regulating the expression of SERPINB2 gene and SMAD4 transcription factor. Thus, our study is suggestive of certain miRNAs and unexplored pathways which may pave a way to unravel critical therapeutic targets in asthma.

## Introduction

Despite recent advances in anti-asthmatic therapeutics, asthma is still a major global health concern. According to the latest Global Asthma Report 2018, over 339 million people are affected globally by asthma leading to more than 1000 deaths per day (https://globalasthmareport.org). At least 2 billion individuals worldwide have exposure to the contaminated smoke of biomass fuel, usually burned inadequately in fire replaces or poorly ventilated indoor stoves. A billion individuals inhale polluted outdoor air, and a billion have exposure to tobacco smoke^1^. Asthma is a chronic disorder of airways, which is characterized by inflammation resulting from a complex interplay of diverse pathways in numerous type of cells including epithelial cells, smooth muscle cells, neutrophils, mast cells, eosinophils, T cells, and B cells^2,3^. Cellular and molecular processes in asthma are relatively unknown due to heterogeneity in genetic, clinical and treatment responses in asthma subjects. Airway re-modelling takes places in severe asthma characterized by goblet cell metaplasia, epithelial damage, sub-epithelial fibrosis, basement membrane thickening, and escalated airway smooth muscle^4,5^. Based on phenotypes, there can be non-allergic asthma, allergic asthma, asthma with obesity, adult-onset asthma, and asthma with persistent airflow limitation^6,7^ whereas based on the severity it could be mild, moderate or severe. Molecular phenotyping of diseased tissues helps to identify biomarkers of asthma which are essential for the development of more localized and molecularly targeted therapies based on the heterogeneity of patients^8^.

There are two subgroups of asthma based on the expression of Th2 associated genes. One subgroup expresses a higher level of Th2 associated genes. While another subgroup which lacks few Th2 associated genes in airways epithelial cells is termed as non-Th2 associated. This non-Th2 asthma group may be low-Th2 asthma/ Th1 driven asthma. In Th2 group asthma patients, CD4^+^ T cells produce IL-4, IL-5, and IL-13 (Th2 cells), which have been reported in bronchoalveolar lavage and airway autopsies. There has been an anticipation that asthma is resulted due to an inflammatory response stimulated by Th2 cells. Increment of Th2 lymphocytes in the airways of allergic asthmatic patients occurs after antigen challenge. These are the cytokines which have been usually reported in mild or asymptomatic asthma^9^. IL-13 and IL-4 are the potent activators of antibody production by B cells especially IgE, while IL-5 is crucial for eosinophil maturation and differentiation. Although there are no unanimously accepted biomarkers for Th2 asthma group, they are characterized by the atopy, eosinophilic inflammation, and a good clinical response to inhaled corticosteroids (ICSs)^10^. The non-Th2 asthma group is usually less responsive to ICSs. The non-Th2 asthma group includes asthma driven by smoking, pollutants, viral/ bacterial infections or obesity, late-onset asthma, etc. Low Th2 asthma can be due to many cellular signalling pathways, including neutrophilic inflammation by dysregulation of the Th17 pathway, oxidative stress, Th1 mediated processes which may include a Th2 component also. Th1 cells were identified in many non-Th2 patients’ airways which play a role in the pathogenesis of the disorder. Study of IFN-γ production in the patients indicated that Th1 cells in asthmatics are not allergen-specific and these may be either bystander in airways or must be playing some regulatory capacity role^11,12^.

The primary objective of the research is to pinpoint the Differentially Expressed Genes (DEGs) between asthmatics and controls from mRNA expression profiles of epithelial cells lining Nasal and Bronchial airway from two different studies to strengthen the statistical analysis. Pathway and Gene Ontology (GO) term enrichment analysis of these DEGs helps to uncover which pathways are dysregulated in asthma. Protein–Protein Interaction (PPI) network was also formed which helped in deciphering the underlying molecular mechanism in the pathophysiology of the disease. Subsequently, hub genes were elucidated from the PPI network by taking into consideration some of the vital network centrality measures. Finally, driver microRNAs (miRNAs) and Transcription Factors (TFs) associated with these asthma-associated DEGs are identified via Feed-Forward Loop (FFL).

In accordance with graph theory, the basic information of the PPI network is provided by its centralities. The blend of PPI centrality measures and important biological knowledge acts as a mean for identifying the biological mechanism of species^13^. Node degree centrality, closeness centrality, betweenness centrality, stress centrality, and EPC are the essential measures in network theory that have been used for detecting effective connections in biological networks. Recently, centrality-based measures for hub genes identification have been applied to many networks such as PPI networks, gene co-expression networks, and cancer metabolism networks^14^. Ernesto Estrada et al*.*^15^ showed that subgraph-based six centrality measures overpowered classic measures for identifying vital proteins in a PPI network. Jeong et al*.*^16^ exhibited that nodes with high degree centrality in a yeast PPI network correspond to crucial proteins.

The DEG biomarkers identified using computational approach are comprehensively regulated by TFs and miRNAs. TFs are cis-regulating molecules which act on gene’s promoter region thereby regulating gene transcription. On the other hand, miRNAs are the small (20–22 nucleotide) non-coding RNA molecules which regulate gene expression post-transcriptionally. miRNAs exert their control by acting post-transcriptionally by binding in 3′ UTR of the gene. They are known to exert epigenetic control over various cellular process like cell division, differentiation, apoptosis, disease progression, etc.^17^. TFs and miRNAs act in an intricate regulatory network which is tightly coupled to form a common regulatory logic for gene regulation^18^. In our study, we utilized microarray-based mRNA expression profiles of bronchial and nasal airway samples of asthmatic and control patients. Both nasal and bronchial airway epithelium is composed of ciliated, basal, and secretory epithelial cells^19^. The significant regulatory networks identified from microarray-based expression profiling of bronchial and nasal airway can be combined to form a transcriptional/ post-transcriptional FFL. In this study, we pursued a regulatory network-based approach to identify critical miRNAs, DEGs and TFs which form asthma specific 3-node miRNA FFL network. Study of these regulatory FFLs may lead to the identification of specific biological events and mechanisms which are responsible for determining cell fate in asthma and other allergic diseases. Identification of critical regulatory genes, TFs, and miRNAs targeting the Th2 inflammatory pathway or the altered gene expression of the bronchial or nasal airway in asthma patients would be a valuable tool in elucidating pathogenesis and therapeutic strategies in asthma.

## Results

### Microarray data extraction and preprocessing

Based on the inclusion and exclusion criteria specified in the methods section, microarray gene expression profiles with accession numbers GSE41861 and GSE41862 were downloaded. GSE41861 contains expression data from Bronchial Samples (BS) and Nasal Samples (NS) of 91 asthmatic patients and 47 controls, respectively. And, GSE41862 contains expression data from BS of 95 asthmatic patients and 21 controls, respectively. Information related to these datasets like GEO accession number, platform type, number of samples, type of study, and species is shown in Supplementary Table S1, respectively. The intensity scatter plots for visualizing the variance in datasets GSE41861 and GSE41862 are shown in Supplementary Figure S1.

### Meta-analysis and identification of DEGs

**Figure 1:**
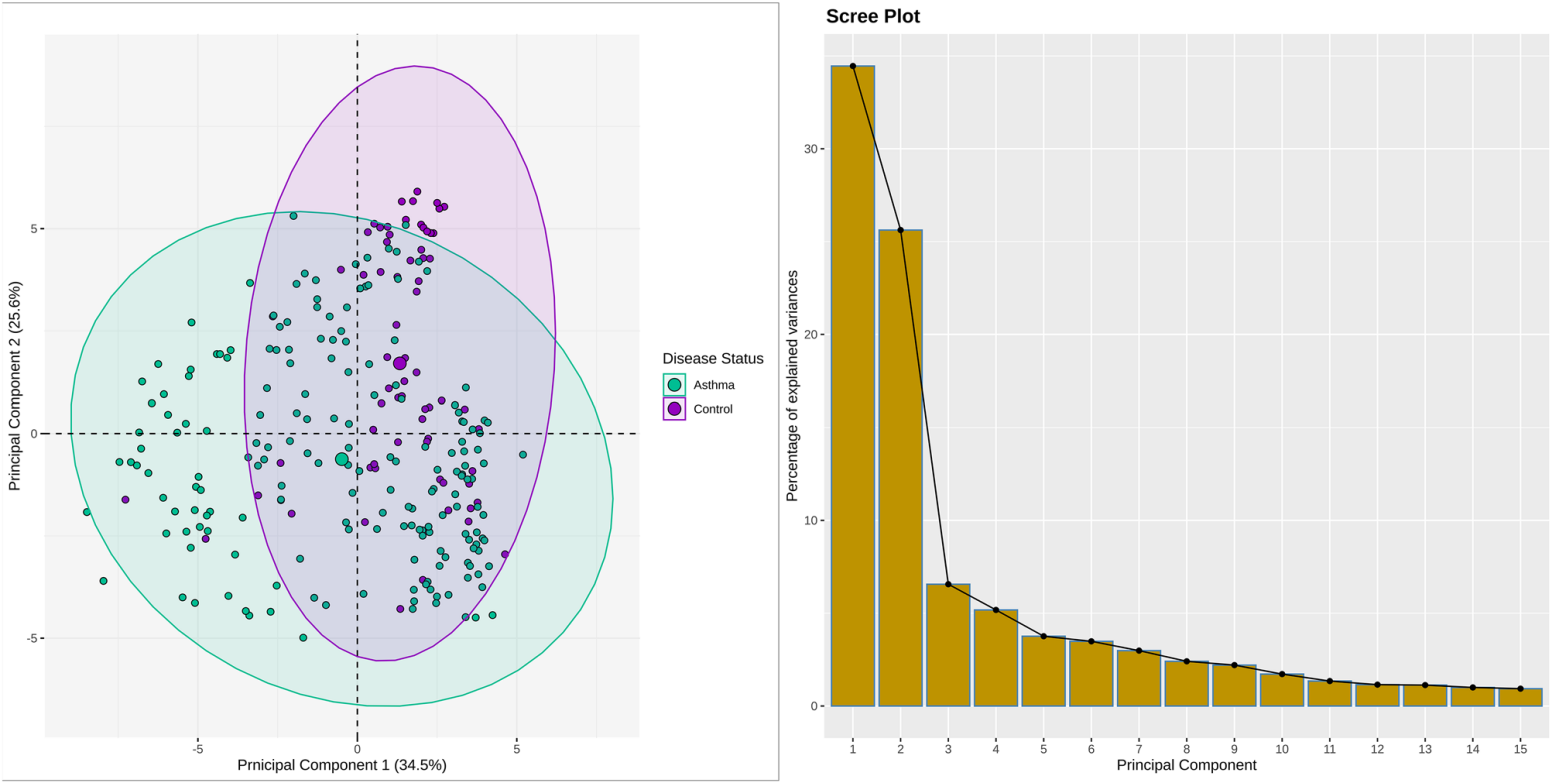
PCA plot in the left panel represents the variation in the expression data between controls and asthmatics. Each point in the plot shows the overall expression value of 30 screened DEGs. The color of each point represents the disease status: magenta for controls and green for asthmatics. The percentage of total variation which is accounted for by the 1st and 2nd principal components are shown on the x and y axes respectively. Scree plot in the right panel shows the percentage of explained variances captured by their corresponding principal components.

**Figure 2:**
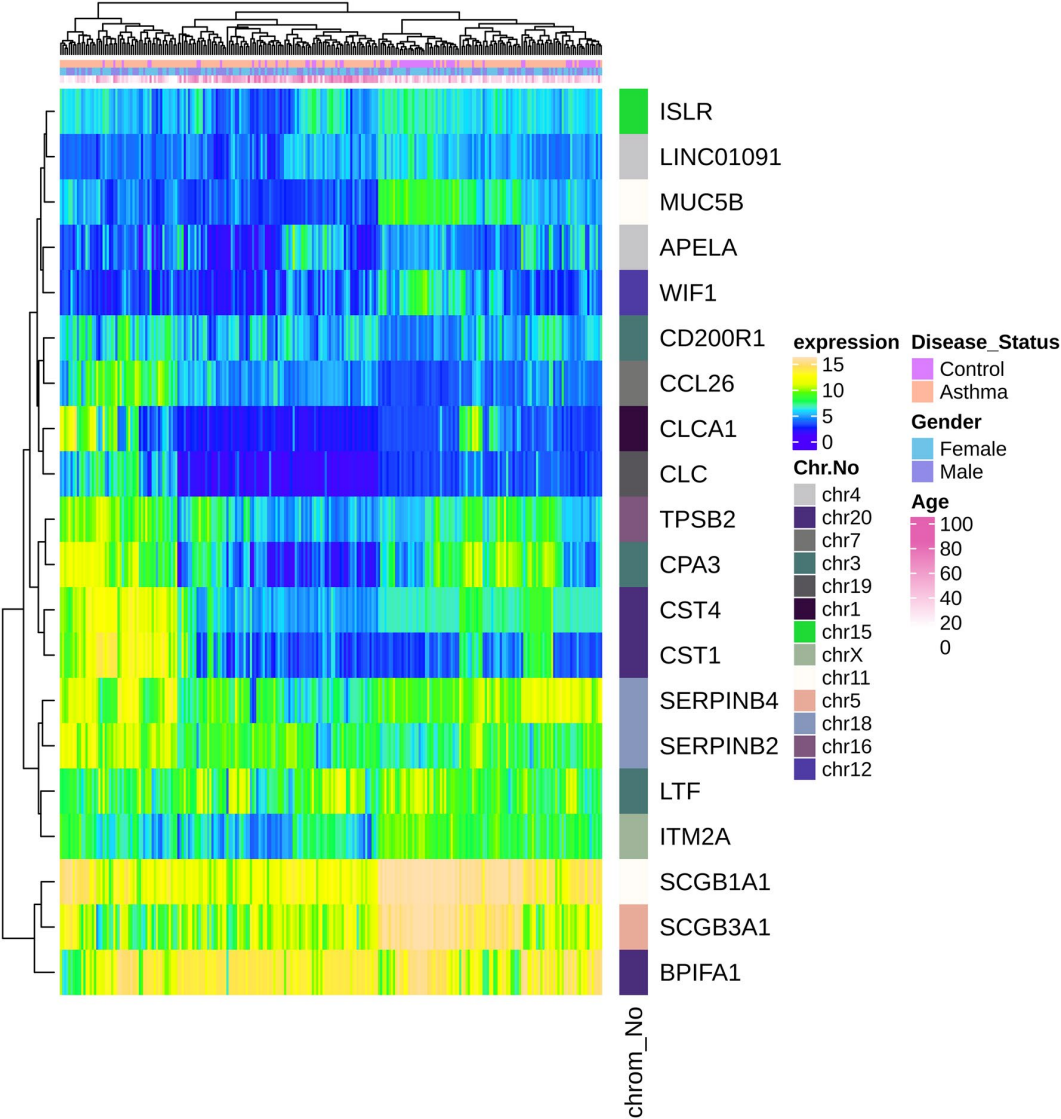
Heatmap plot of top 10 up and downregulated DEGs in asthma. Expression values for each DEG (row) are normalized across all the samples (columns). Hierarchical clustering using Euclidean distances was employed for both rows and columns with cluster dendrograms presented along the left and top sides of the plot. The categorical annotation bars (above the heatmap) show the column annotations for age, gender and disease status. The location of each gene on its respective chromosome was shown in the right bar (color bands) as row annotation.

A total of 30 DEGs were yielded after meta-analysis of microarray datasets involving asthmatics vs controls and applying the statistical threshold of *BH*-*p*-value and log_2_ (fold change) respectively. Figure 1 shows the Principal Component Analysis (PCA) plot (left panel) representing the variation and clusters of 30 asthma-associated DEGs across 2 dimensions, while the scree plot (right panel) represents the percentage of variances accounted for by the principal components, respectively. Among the total DEGs, 18 were upregulated and 12 were downregulated based on the log_2_ (fold change) criterion. Annotated heatmap of top 10 up and downregulated DEGs is shown in Fig. 2.

### Pathway & GO term enrichment analysis

**Figure 3:**
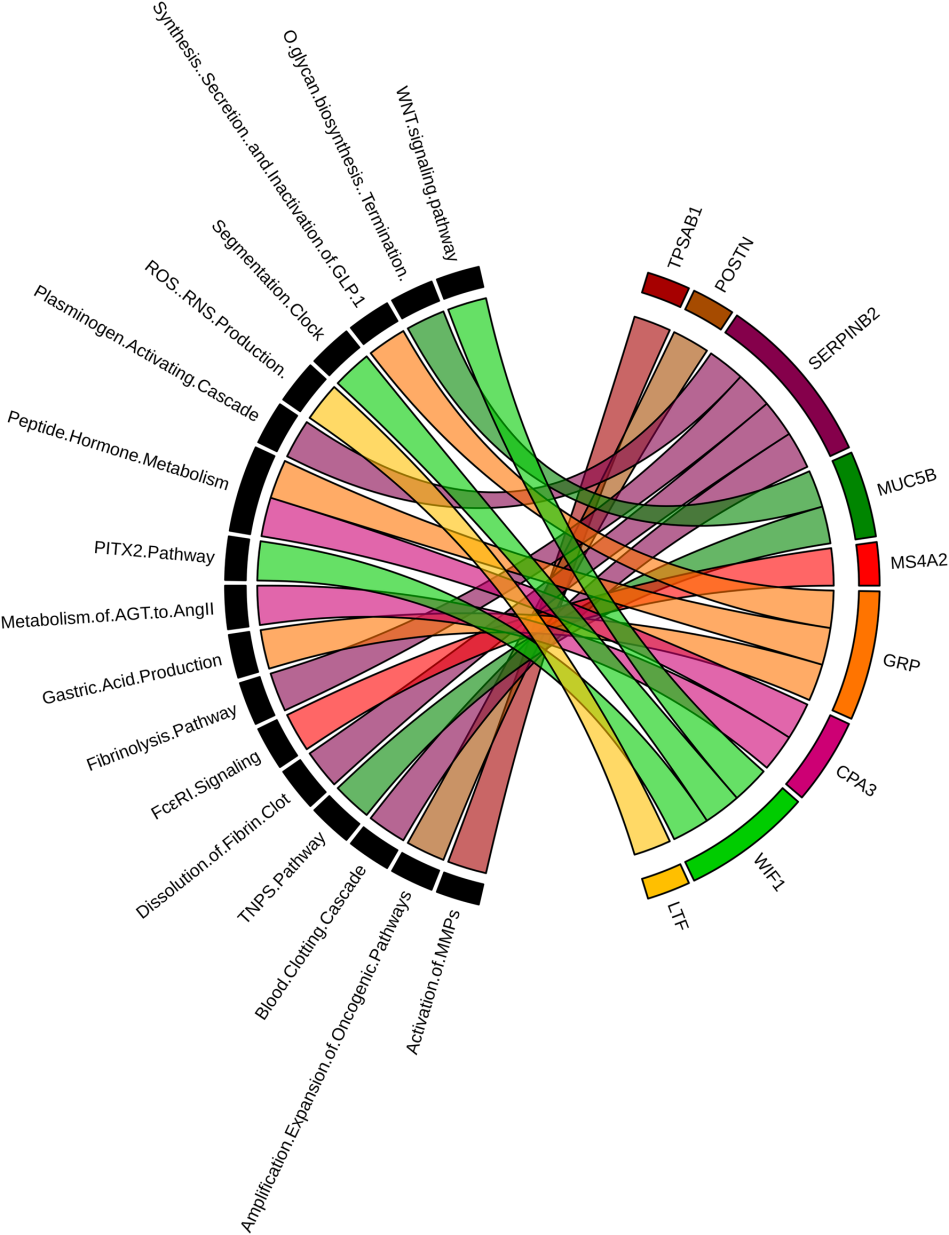
Circos plot representation of significantly enriched pathways linked with 9 asthma-associated DEGs. Outside the circle, 17 significantly enriched pathways (on the left) and 9 dysregulated genes (on the right) are indicated. Each DEG is denoted by a unique color band and the undirected colored edge inside the circle represents the association of a particular gene with their respective pathway(s).

A total of 13 DEGs out of 30 were involved in significantly enriched pathways and GO terms. Nine DEGs (MUC5B, POSTN, SERPINB2, CPA3, GRP, LTF, MS4A2, TPSAB1, and WIF1) were involved in a total of 17 significantly enriched pathways (*BH*-*p*-value < 0.05) with peptide hormone metabolism being the most significant pathway (*BH*-*p*-value = 0.006547577) and ROS, RNS production in response to bacteria being the least significant pathway (*BH*-*p*-value = 0.04979756). A circos plot showing the association of 9 DEGs with 17 significantly enriched pathways is shown in Fig. 3. Six DEGs (SERPINB10, SERPINB4, SERPINB2, LTF, CST1, and CST4) were involved in significantly enriched GO-Biological Process (BP) term: negative regulation of endopeptidase activity (GO:0010951) and GO-Molecular Function (MF) terms: endopeptidase inhibitor activity (GO:0004866), serine-type endopeptidase inhibitor activity (GO:0004867), and cysteine-type endopeptidase inhibitor activity (GO:0004869) respectively (Supplementary Table S2).

### PPI network analysis and hub gene identification

**Figure 4:**
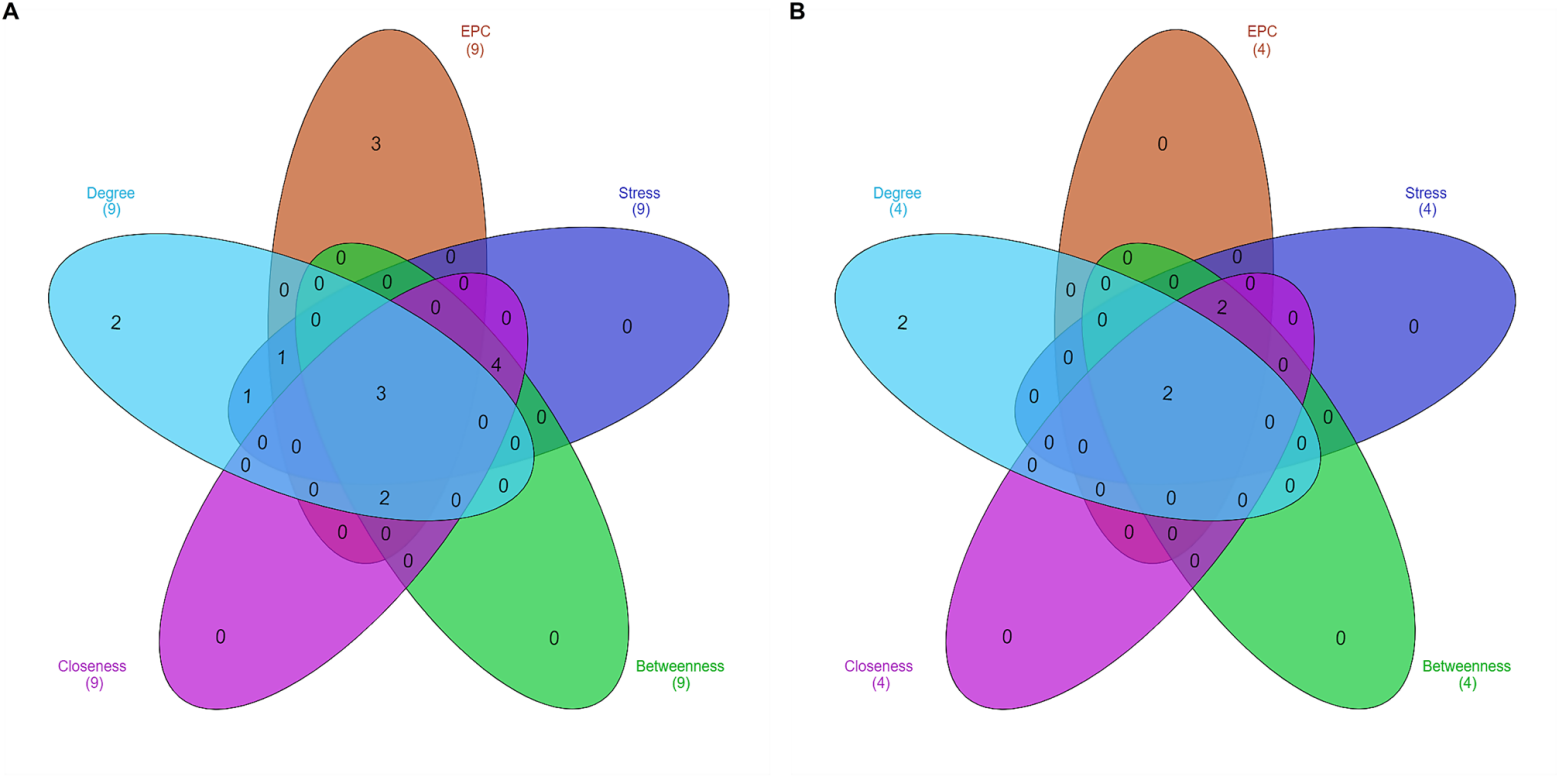
Venn plots showing the significant hub genes obtained from (**A**) upregulated and (**B**) downregulated PPI networks, respectively. Areas with different colors correspond to different centrality measures. The cross-intersection area shows the hub genes in both networks. A total of 5 hub genes were obtained for the up and downregulated PPI networks.

**Figure 5:**
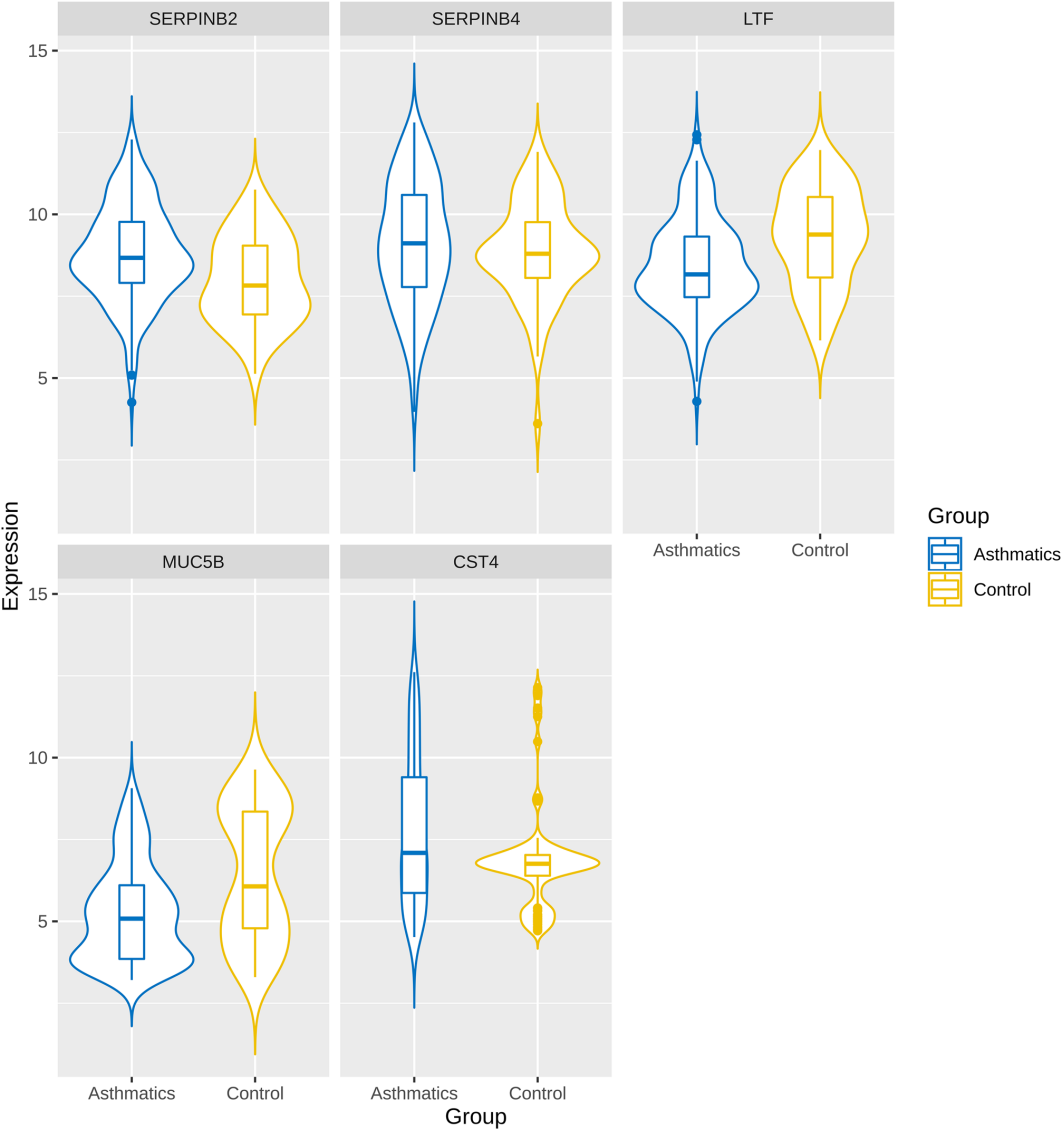
Violin plots of 5 hub genes filtered based on functional enrichment analysis and PPI network centrality measures respectively. This plot represents the distribution density of the underlying expression data for hub genes. The top and bottom of the embedded box signify the 75th and 25th percentile of the distribution, respectively. The line inside the box represents the median. Endpoints of the axis are labelled by the minimum and maximum values. Control and asthmatic samples are distinguished by yellow and blue colors, respectively.

Unique 13 DEGs (9 upregulated and 4 downregulated DEGs) obtained after enrichment analysis were further used for the PPI network construction and analysis using Cytoscape. Both up and downregulated PPI networks are shown in Supplementary Figures S2 and S3. The topological properties of up and downregulated PPI networks are shown in Supplementary Table S3 respectively. Top n functionally enriched DEGs (*n* = 9 for upregulated & *n* = 4 for downregulated) were ranked based on the centrality measures. A total of 5 intersecting hub genes (3 upregulated, i.e., CST4, SERPINB2, SERPINB4 and 2 downregulated, i.e., LTF, MUC5B) were obtained and shown by the Venn plots in Fig. 4A and B respectively. Violin plots showing the expression distribution of these hub genes in controls and asthmatics are shown in Fig. 5 respectively. Among all the hub genes, CST4 and LTF were most upregulated and downregulated across asthmatic samples.

### Asthma-specific miRNA FFL regulatory network

**Figure 6:**
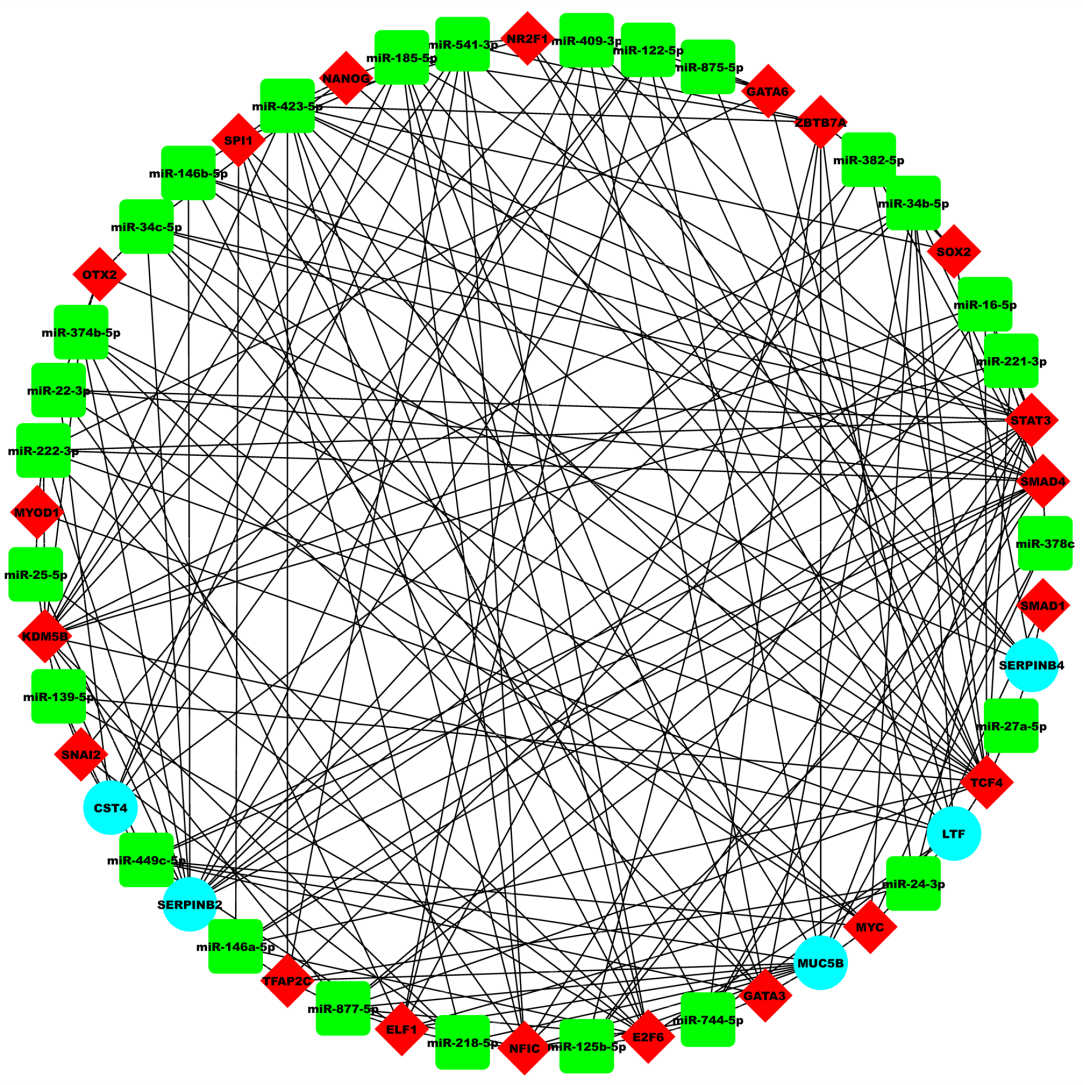
Asthma-specific 3-node miRNA FFL regulatory network with 51 nodes and 197 edges, respectively. The green-colored rectangular nodes represent asthma-associated miRNAs, red-colored triangular nodes represent transcription factors, and cyan-colored circular nodes represent asthma-associated hub genes.

**Figure 7:**
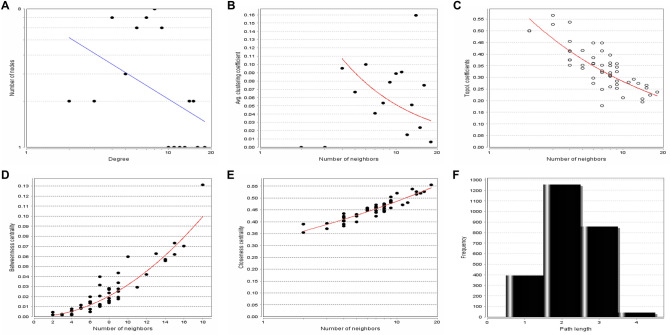
Topological parameter graphical plots of the asthma-specific 3-node miRNA FFL representing (**A**) Node degree distribution, (**B**) Average clustering coefficient, (**C**) Topological coefficient, (**D**) Betweenness centrality, (**E**) Closeness centrality, (**F**) Shortest path length distribution. The lines are fitted with power laws.

The 3-node miRNA FFL involved a total of 51 unique nodes and 197 edges (Fig. 6). Among these edges, 32 belonged to miRNA-gene pairs, 21 to TF-gene pairs, and 144 to miRNA-TF pairs as shown in Table 1. Amongst the 51 nodes, 5 belonged to asthma-associated hub genes, 26 to asthma-associated miRNAs and 20 to asthma-associated human TFs. Different topological components, including the distribution of node degree, average clustering coefficient, topological coefficient, betweenness centrality, closeness centrality, and shortest path length for the miRNA FFL network are shown in Fig. 7A–F respectively. The node degree distribution for miRNAs, TFs, and genes in the miRNA-FFL was right-skewed, depicting that majority of nodes had a low degree, with only a minor section of nodes having a high degree. Most strongly connected network motif or subnetwork in our FFL contained one TF (SMAD4), one hub gene (SERPINB2), and two miRNAs (miR-449c & miR-34b-5p).

**Table 1:** Summary of relationships among Asthma-associated DEGs, Asthma-associated miRNAs, and TFs.

Relationship	No. of edges	No. of miRNAs	No. of TFs	No. of genes
miRNA-gene^a^	32	26	-	5
TF-gene^b^	21	–	20	5
miRNA-TF^c^	144	26	20	–

^a^miRNA-gene: miRNA repression of genes.

^b^TF-gene: TF regulation of genes.

^c^miRNA-TF: miRNA repression of TFs.

#### miRNA-gene and miRNA-TF repression

A total of 26 miRNAs targeted the five hub genes. SERPINB2 was repressed by maximum miRNAs (i.e.,13). Also, both miR-34b-5p and miR-449c-5p targeted highest number of genes (i.e.,3). miRNA-TF interaction had 144 edges between 26 miRNAs and 20 TFs respectively. TCF4 was repressed by the highest number of miRNAs (i.e., 17). miR-423-5p repressed highest number of TFs (i.e.,12).

#### TF-gene regulation

A total of 20 TFs regulated the five hub genes where both LTF and MUC5B were regulated by a maximum number of TFs (i.e., 7). Also, NR2F1 targeted the highest number of hub genes (i.e., 2).

## Discussion

Transcriptional regulatory networks are the key functional motif networks which interplay in various diseases including asthma leading to a molecular pathological outcome. During recent years, databases of interaction prediction, verification of experimental methods and confirmation of loops have been continually emerging^20^. The integrated results of five important PPI-based centrality measures (EPC, degree, stress, betweenness and closeness) leading to vital hub genes resolve the outcomes which were inconsistent as provided by different other methods^21^. In our study, we identified 30 asthma-linked DEGs after the meta-analysis of microarray datasets and deduced 13 functionally enriched DEGs which formed the two backbone PPI networks. From these two networks, 5 hub genes were found based on their degrees and other centrality measures. The upregulated genes being CST4, SERPINB2 and SERPINB4, while downregulated genes being LTF and MUC5B, respectively. Asthma-associated miRNAs and TFs having a relationship with these hub genes were also discovered. In our study, we utilized key transcriptional level interaction i.e. miRNA-gene repression, miRNA-TF repression, and TF-gene regulation for the construction of miRNA centred feed-forward loop which plays a critical role in asthma (Fig. 6). The results of our study identified two key miRNAs, i.e., miR-34b and miR-449c which regulated the SERPINB2 gene and SMAD4 transcription factor, being the part of FFL.

Most of the hub genes observed in our study were involved in protease/antiprotease pathways (SERPINB2, SERPINB4, CST4). These upregulated hub genes are also indicative that neutrophilic inflammation in asthma may be driven by the activated T cells. Cystatin S (CST4), which is an epithelial gene, was found to be a differentially expressed hub gene in our study as also revealed by other research groups in different study cohort and methods^22,23^. Cystatins have recognized roles in inflammation where they act as potent competitive inhibitors of cysteine proteinases (cathepsins) being present in all biological fluids^24^. Induction of synthesis of Tumor necrosis factor-alpha (TNF-α) and IL-10 are among the potent immunomodulatory roles of cystatins. Another hub gene Mucin-5B (MUC5B) is a marker of secretory cells which was downregulated in asthmatic subjects in our study. MUC5B is expressed predominately in the mucous glands. Although our study datasets didn’t define the severity level of asthma, epithelial MUC5B repression was observed in mild-moderate asthma by other studies^23^. Changes in the expression of this gene indirectly reflect the airway remodelling in case of chronic asthma patients^25^.

Lactoferrin or lactotransferrin (LTF) serves as an anti-inflammatory factor and an immunomodulator. LTF which is an iron-binding glycoprotein has been shown upregulated during asthma development in our study as also reported by other studies^26,27^. A report by Lourdes Fernández-Delgado et al*.* suggested a new mechanism of LTF release by human neutrophils on activation by galectin-3 and claimed the pro-inflammatory role of LTF in allergic asthma^28^. LTF could reduce airway inflammation in the pollen-induced allergic mouse model and its allergen mediated release could be correlated to the severity of asthma symptoms^28,29^. Thus, being a hub gene also as elucidated in our study, LTF can be suggested as a putative tool to track the progression or the drug efficacy in allergic asthma patients.

Serine protease inhibitors (SERPIN) genes like SERPINB2 and SERPINB4 have an important role in asthma pathogenesis. SERPINB2 or Serpin B2 prevents plasmin activation by inhibiting plasminogen activators. Plasmin can degrade ECM directly by removal of glycoproteins or indirectly by activation of metalloproteinases^30^. Therefore, by inhibition of SERPINB2, plasmin availability for extracellular matrix turnover increases and reduces airway remodeling. Increased SERPINB2 in airway epithelial cells has been described as a biomarker gene signature for Th2-mediated inflammation in asthma^31^. It is induced by Th2 cytokine interleukin (IL-13) which also acts as the central regulator of goblet cell metaplasia^32^. An increase in mucous cell number (mucous cell metaplasia) and a decrease in ciliated cells number has been linked with epithelial cells stimulation by IL-13^33^. Obstruction of airways by mucus is a key contributor to fatal asthma. As it has been known that changes in airway epithelial cell differentiation, driven in part by IL-13 are essential in asthma, miRNAs are known to regulate cell differentiation in various systems and could contribute to epithelial abnormalities in asthma. SERPINB4 or SCCA2 gene is a potent inhibitor of apoptosis. It can inhibit apoptotic serine proteinases, such as cathepsin G and mast cell chymase which results in the persistence of memory Th2 cells that can give rise to inflammatory lung disease like asthma^34^. It was identified as an upregulated hub gene in our study as also described by other studies^35,36^. Thus targeting SERPINB4 as a localized molecular target leading to depletion of memory Th2 cells can help deplete the reservoir of inflammation in allergic asthma disease.

In asthma patients, TGF-*β* (Transforming growth factor-beta) increases connective tissue growth factor (CTGF) expression in airway smooth muscle cells which leads to deposition of extracellular matrix proteins, fibronectin, and collagen I^37^. Wang et al*.*^38^ revealed a TGF-*β*-SMAD4 and SMAD2 mediated epigenetic regulatory pathway for IL-9 production by differentiated Th9 cells. IL-9 is a proallergic cytokine which plays a key role in asthma induction. SMAD proteins may displace EZH2 directly from the Il9 locus by binding to it leading to IL-9 expression. The probability of cells to exhibit a sustained CTGF transcriptional response was increased by high total cellular SMAD4 but not SMAD2 levels^39^. These studies pointed out the critical role of SMAD4 in TGF-*β* mediated signaling in asthma^40^. TGF-*β*-SMAD4 axis may be, therefore, explored as a novel therapeutic target to increase bronchodilators (mainly *β*_2_-adrenoceptor agonists) sensitivity in severe asthma. Our studies pointed out SMAD4 as a critical transcriptional factor involved in regulatory aspects of asthma pathogenesis. During fungal protease-mediated epithelial inflammation, downregulation of Uncoupling Protein 2 (UCP2) expression by regulatory TGF-*β*-SMAD4 signaling was associated with mitochondrial reactive oxygen species (ROS) production^41^.

Based on our analysis, we identified mir-34b-5p and miR-449c-5p (members of the miR-34/449 family) as highest-ranked microRNAs involved in the regulation of FFL involving transcription factor SMAD4 and SERPINB2 hub gene in asthma. Here based on the study it can be hypothesized that in asthma repression of miR 34b/449 regulates upregulation of SERPINB2 mediated via SMAD4 upregulation by an unknown direct or indirect regulatory process. Although this needs further validation to confirm the molecular mechanism underlying this. Although a few studies have been done to explore the role of miR-34b/ 449 in asthma but all of them indicate the key pathological role of miR 34b/ 449 in inflammation. An important study done by Solberg et al*.*^42^ shows that the miR-34/449 family members (miR-34c-5p, miR-34c-5p, miR-449a, and miR-449b-5p) are repressed significantly in vivo in asthma, in vitro by IL-13 exposure, and even after corticosteroid treatment. This repression of miRNA levels was induced by IL-13 resulting in increased notch expression as well as mucous cells increase in asthma. miR-449 acts as a crucial regulator of differentiation of nasal epithelial cells to ciliated cells via repression of transcript levels of NOTCH1 and its ligand Delta-1 (DLL1)^42,43^. miR-34/449 also modulated Insulin-like growth factor-binding protein 3 (IGFBP-3) mediated autophagy activation in lung epithelial cells in vitro^44^. Thus our study may provide an important link on miR-34b and miR-449 mediated regulation of Th2 cell signature gene SERPINB2 via IL-13. Given the fact that steroids have very little effect on miR expression in asthma, our study may help identify therapeutic targets which may lead to synchronized control in Th2-mediated inflammation. The study points out the possible role of 3′,5′-cyclic adenosine monophosphate (cAMP) signaling pathways via SMAD4 as a critical regulator in asthma inflammation. This may be further mediated by increased PDE4 (Phosphodiesterase isomer) subsequently resulting in decreased Th2 biomarker like SERPINB2. miR-34b and miR-449 may be potential therapeutic targets which exert pathological inflammation by SMAD4 and SERPINB2. Exerting control by these two differentially expressed miRNAs can result in control in pathology exerted by airway epithelial cells in asthma.

The results obtained from our study exhibited the differential expression of hub genes along with their related mechanisms that could be used as potential therapeutic targets for asthma. Each hub gene and its respective partner may assist in gaining clear insights into the inflammation progress in mild, moderate, and severe asthma. Thereby, the differential expression pattern of hub genes reported in our study might help to gain insight into many unexplored regulatory pathways leading to unravelling the critical targets for increasing drug efficacy, diagnosis, and prognosis in asthma.

## Methods

### Data collection and preprocessing

Microarray datasets containing mRNA expression profiles for asthmatics and controls were downloaded from NCBI Genome Expression Omnibus (GEO)^45^. The keyword “asthma” was used for this search. As per the Preferred Reporting Items for Systematic Review and Meta-Analysis (PRISMA) guidelines^46^, the eligible studies and datasets should follow these inclusion criteria: (i) having patient and control human samples, (ii) expression profiling by array type, (iii) having complete raw and processed microarray tissue data, (iv) submitted in year range of 2015 to 2020, and (v) derived from the same microarray platform. Review articles, abstracts, case reports, studies without healthy control or non-human samples, and cell-line based experimental study designs were excluded. GEOquery package^47^ in R was used to download the CEL files which contained unprocessed mRNA expression profiles. Unprocessed expression data from CEL files were read as an affy-object using the affy module^48^ followed by background correction, quantile normalization, and log_2_ transformation using Robust Multiarray Average (RMA) method in R. Also, the probe IDs were mapped to their corresponding HGNC (HUGO Gene Nomenclature Committee) gene symbol(s)^49^ using hgu133plus2.db package available in R. Relative expression values across genes mapping to more than one probe IDs were averaged^50,51^.

### Meta-analysis of microarray datasets

The meta-analysis of the normalized gene expression datasets was performed in R using metaMA^52^ and limma^53^ packages respectively. These packages employed functions for t-test and combined *p *value computation. The combined *p *value algorithm is based on Fisher’s sum of logs method^54^ and sums the logarithm of *p *values across k-studies by combining datasets across samples for the same gene. The *p *values were adjusted using False Discovery Rate (FDR) method as given by Benjamini and Hochberg (BH)^55^. Combined *p *values were computed for the common genes between both the datasets followed by their BH-correction. The DEGs between asthmatic and control samples were screened at *BH*-*p*-value < 0.05 and an absolute log_2_ (fold change) > 0.5. The sets of significant genes corresponding to a log_2_ (fold change) > 0.5 and log_2_ (fold change) < -0.5 were categorized as the up and downregulated DEGs, respectively.

### Pathway and GO term enrichment analysis

Pathway enrichment analysis was performed by different Enrichr^56,57^ gene set libraries like BioCarta, Reactome, Panther, NCI-Nature, and WikiPathways, respectively. To account for multiple comparisons, the pathways with *BH*-*p*-value < 0.05 were considered as significantly enriched. And, the GO term enrichment analysis was performed by Enrichr gene set libraries like GO-BP and GO-MF respectively. Terms with *BH*-*p*-value < 0.05 were considered as significantly enriched. Union of all DEGs involved in significantly enriched pathways and GO terms were used for further analysis.

### PPI network analysis and hub gene identification

Unique DEGs obtained after enrichment analysis were used for PPI network construction and analysis. Interacting human protein partners of these DEGs were retrieved using the Biological General Repository for Interaction Datasets (BioGRID v3.5) (https://thebiogrid.org/)58 and Human Integrated Protein–Protein Interaction rEference (HIPPIE v2.2) (https://cbdm-01.zdv.uni-mainz.de/~mschaefer/hippie/)59 databases respectively. The protein interactors having at least one reported experimental evidence were selected from BioGRID. And, the protein interactors with 0.63 ≥ *score* ≤ 1 (corresponding to medium and high confidence interactions) were selected from HIPPIE**.** Both up and downregulated PPI networks were analyzed using the *NetworkAnalyzer* plugin available in Cytoscape v3.7.2^60^. The *CytoHubba* plugin available in Cytoscape was used for hub genes identification^61^. Top n DEGs (where n is the number of DEGs in up and downregulated PPI networks) were ranked based on the five characteristic centrality measures like degree, stress, closeness, betweenness and EPC using CytoHubba. Intersecting DEGs from these five centralities for both the PPI networks were considered as the hub genes respectively.

### Mining of significant interaction pairs between miRNAs, TFs, and hub genes

#### miRNA-gene repression

Retrieval of miRNA-gene pairs was performed using miRWalk v3.0^62^, miRSearch v3.0^63^ and Starbase v2.0^64^ databases respectively. Binding gap = 1, 3UTR region and score ≥ 0.95 were regarded as the threshold criteria for significant miRNAs selection in miRWalk respectively. Significant miRNAs having a high score denoted by the green-colored band were retrieved using miRSearch.

#### TF-gene regulation

TF-gene pairs retrieval was performed using ChIPBase v2.3 database^65^, ITFP database^66^, and Enrichr platform gene set libraries like ChEA, TRANSFAC, and JASPAR PWMs. TFs with binding sites location within 1 kb upstream region were extracted from ChIPBase. And, significant TFs with *p *value < 0.05 were extracted from Enrichr libraries.

#### miRNA-TF repression

The TFs obtained from TF-gene interaction were further utilized for fetching the relevant miRNA-TF pairs. TFs targeted by miRNAs were extracted by miRWalk, miRSearch, and Starbase databases respectively with the same thresholds described previously.

We required that the miRNA-target interactions be evolutionarily conserved in human and mouse. For this, mouse miRNAs targeting the available human TFs and genes were also retrieved using the same databases as described for miRNA-TF/gene interactions respectively. miRNAs common between human and mouse were considered as highly significant and validated (final miRNAs). All the three types of regulatory interactions were altered with respect to these final miRNAs. These molecular interactions were then merged to form a 3-node miRNA FFL^67^ and subsequently visualized using Cytoscape.

## Supplementary information

Supplementary Information.
