# Supplementary material for: Transcriptomic analysis delineates potential signature genes and miRNAs associated with the pathogenesis of asthma
Source: Sci Rep. 2020 Aug 7;10:13354. doi: 10.1038/s41598-020-70368-5 (PMC7414199; doi:10.1038/s41598-020-70368-5)
Supplement: Supplementary file 1 — Supplementary Information. [file 41598_2020_70368_MOESM1_ESM.docx]

**Transcriptomic analysis delineates potential signature genes and miRNAs associated with the pathogenesis of asthma**

**Prithvi Singh^1^**^,^**^#^**, **Archana Sharma^2^**^,^**^#^**, **Rishabh Jha^1^**, **Shweta Arora^2^**, **Rafiq Ahmad^3^, Arshad Husain Rahmani^4^**, **Saleh A. Almatroodi^4^**, **Ravins Dohare^1^**^,^**^*^**, **Mansoor Ali Syed^2^**^,^**^*^**

**^1^** Centre for Interdisciplinary Research in Basic Sciences, Jamia Millia Islamia, New Delhi 110025, India

**^2^** Translational Research Lab, Department of Biotechnology, Faculty of Natural Sciences, Jamia Millia Islamia, New Delhi 110025, India

**^3^**Centre for Nanoscience and Nanotechnology, Jamia Millia Islamia, New Delhi 110025, India

**^4^** Department of Medical Laboratories, College of Applied Medical Sciences, Qassim University, Buraidah, 51452, Saudi Arabia

**#These authors contributed equally to this work**

**^*^Correspondance:**

Mansoor Ali Syed, PhD Ravins Dohare, PhD

Assistant Professor Assistant Professor

Department of Biotechnology Centre for Interdisciplinary Research in Basic Sciences

Jamia Millia Islamia, New Delhi-110025 Jamia Millia Islamia, New Delhi-110025

E-mail:[smansoor@jmi.ac.in](mailto:smansoor@jmi.ac.in) E-mail: [ravinsdohare@gmail.com](file:///D:\BIOINFORMATICS\SESPSIS%20FINAL\ravinsdohare@gmail.com)

Tel: +91-9953786440 Tel: +91-11-2698-3409

|  | Dataset 1 | Dataset 2 |
| --- | --- | --- |
| GEO Accession No. | GSE41861 | GSE41862 |
| Type of study | Expression profiling by array | Expression profiling by array |
| Platform type | Affymetrix U133 Plus 2.0 | Affymetrix U133 Plus 2.0 |
| Species | Homo sapiens | Homo sapiens |
| No. of samples | 138 (Asthmatics = 91, Non-asthmatics = 47) | 116 (Asthmatics = 95, Non-asthmatics = 21) |

**Table S1.** Characteristics of asthma-associated individual datasets retrieved from GEO


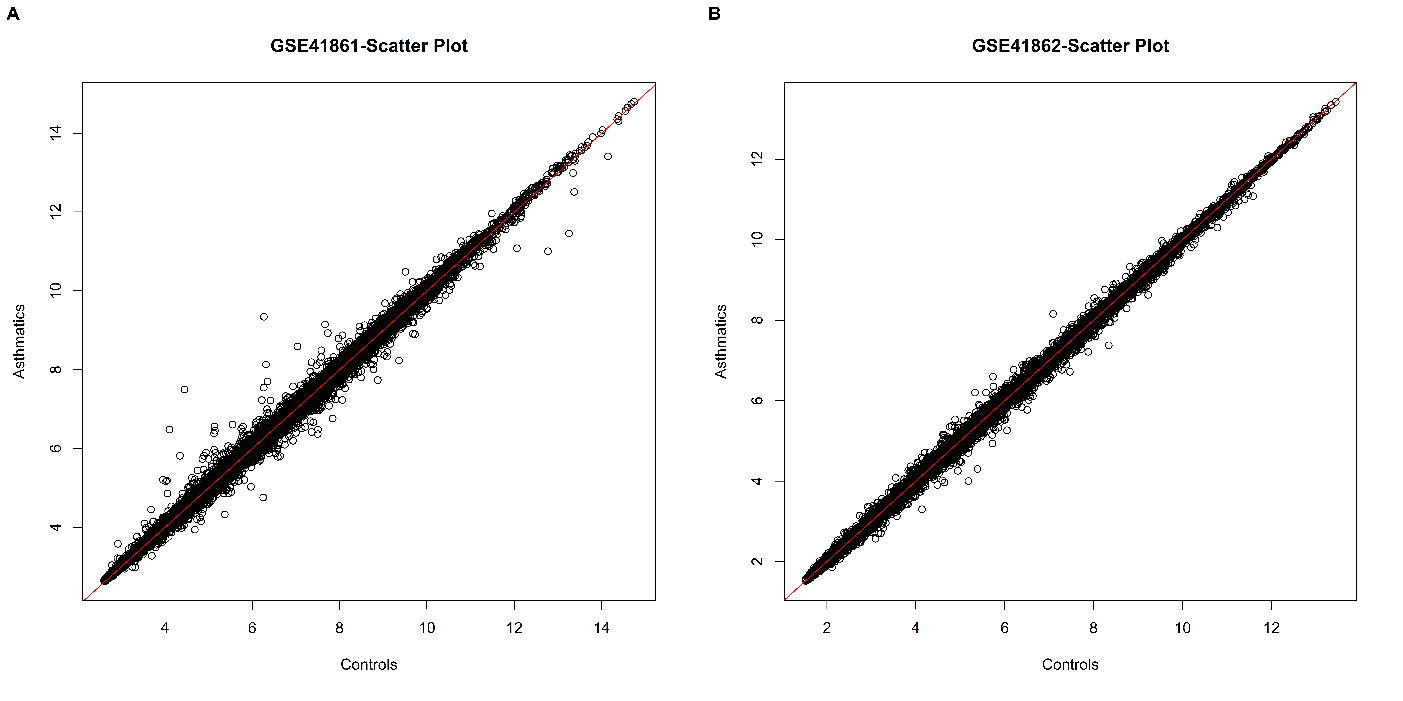


**Figure S1.** Intensity scatter plots showing the relationship between expression values of controls vs asthmatics in datasets (A) GSE41861 and (B) GSE41862, respectively. Each point corresponds to a single gene with its coordinates signifying the mean gene expression across healthy controls (x-axis) and asthmatics (y-axis). A total of 20174 genes were present in both the datasets. The points close to the diagonal correspond to the genes with similar expression level in control and asthmatic groups. Points away from the diagonal signify genes which are differentially expressed in asthmatic samples (upregulated if the points are in upper-half of the plot area and downregulated otherwise).

**Table S2.** GO term enrichment analysis representing the enriched terms (BP and MF) and DEGs involved in them. The significant terms were ranked based on the adjusted p-values.

| **GO Biological Process** | | |
| --- | --- | --- |
|  | **Adjusted p-value** | **Genes involved** |
| negative regulation of endopeptidase activity (GO:0010951) | 1.28E-05 | CST1,CST4,LTF,SERPINB2,SERPINB4,SERPINB10 |
| GO Molecular Function | | |
| endopeptidase inhibitor activity (GO:0004866) | 1.65E-05 | CST1,CST4,LTF,SERPINB2,SERPINB4,SERPINB10 |
| serine-type endopeptidase inhibitor activity (GO:0004867) | 0.032368249 | SERPINB2,SERPINB4,SERPINB10 |
| cysteine-type endopeptidase inhibitor activity (GO:0004869) | 0.036857361 | CST1,CST4,LTF |

**
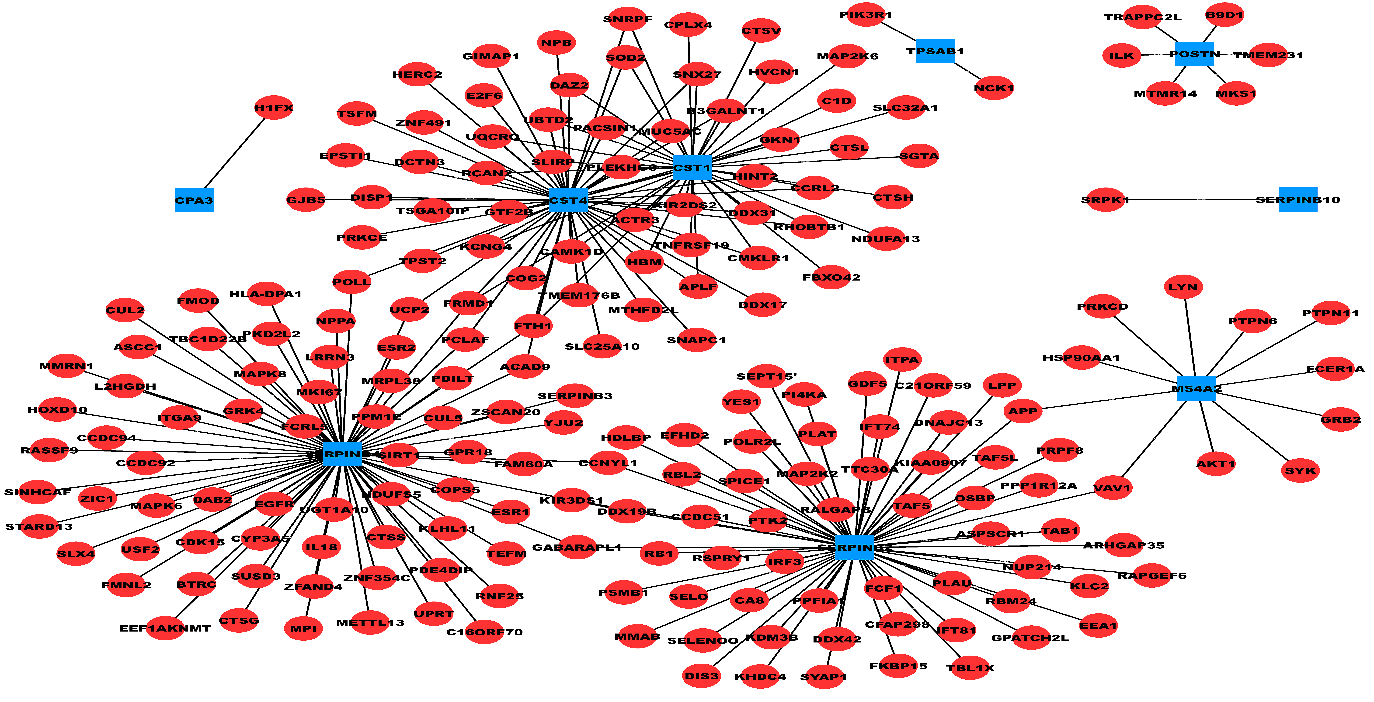
**

**Figure S2.** Backbone PPI network of upregulated DEGs comprising of 212 nodes and 243 edges. The blue colored rectangles represents the 9 upregulated DEGs, the red colored spheres represents their 204 interacting protein partners, and the solid black colored lines represents the edges between them.

**
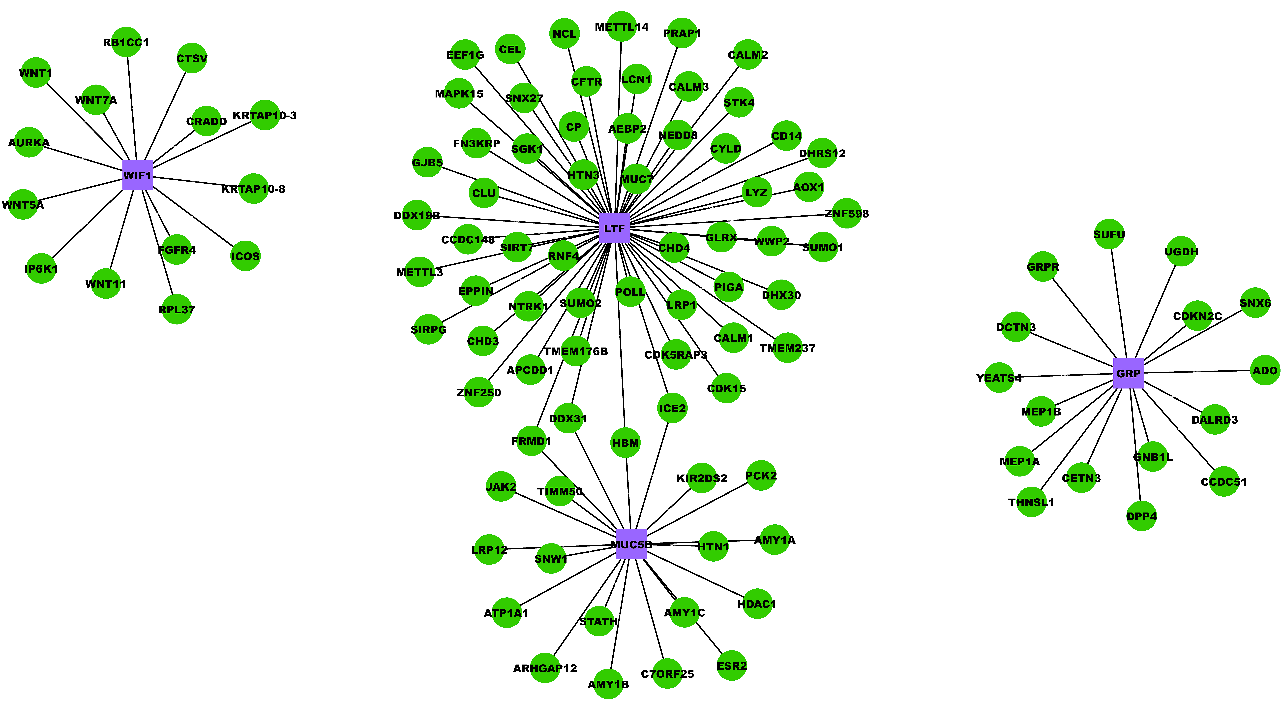
**

**Figure S3.** Backbone PPI network of downregulated DEGs comprising of 106 nodes and 106 edges. The magenta colored rectangles represents the 4 downregulated DEGs, the green colored spheres represents their 102 interacting protein partners, and the solid black colored lines represents the edges between them.

| Topological properties | Values |
| --- | --- |
| Upregulated PPI Network | |
| Number of nodes | 212 |
| Number of edges | 243 |
| Network density | 0.011 |
| Network diameter | 8 |
| Characteristic path length | 3.828 |
| Average number of neighbors | 2.292 |
| Downregulated PPI Network | |
| Number of nodes | 106 |
| Number of edges | 106 |
| Network density | 0.019 |
| Network diameter | 4 |
| Characteristic path length | 2.553 |
| Average number of neighbors | 2.0 |

**Table S3.** Topological properties of the up and downregulated PPI networks.
